# Transurethral ureteric embolization for locally advanced cervical cancer with uretero-vaginal fistula – A case report

**DOI:** 10.1016/j.ijscr.2023.107944

**Published:** 2023-02-18

**Authors:** H. Acton, R. Motyer, J. O'Mahony, I. Brennan, J.M. Ryan

**Affiliations:** Department of Interventional Radiology, St. James's Hospital, Dublin, Ireland

**Keywords:** Embolisation, Uretero-vaginal fistula, Cervical cancer, Urinary diversion, Interventional radiology, Case report

## Abstract

**Introduction:**

Targeted radiotherapy, surgery, and localised disease progression can all result in fistulous tract formation in patients with a pelvic malignancy, in particular cervical or endometrial cancer. This report discusses a novel technique for palliative management of ureterovaginal fistulas in end-stage disease.

**Presentation of case report:**

We report the case of a 37 year old female with metastatic cervical squamous cell carcinoma previously treated with chemoradiation who presented with progressive disease and secondary development of a symptomatic ureterovaginal fistula.

**Discussion:**

This case report discusses the causes and sequalae of uretero-vaginal fistula formation, the role of the interventional radiology with regards to palliative intervention, and potential patient factors that can affect performance of such procedures.

**Conclusion:**

Interventional radiology plays an important role in palliative and symptomatic management of end stage malignant disease. Ureteric embolisation via a retrograde transurethral approach by way of an existing stent is a novel approach to access making the procedure easier for both the patient and radiologist.

## Highlights

• Fistula formation is a common complication of advanced pelvic malignancy.
• Radiological intervention is an important consideration when managing patient symptoms.
• Utilisation of existing devices and patient positioning should be considered to improve the success of the procedure.

## 1 Introduction

Localised pelvic fistulisation in advanced malignancy is not an uncommon finding in patients with gynaecological cancers. Fistulation involving gynaecological malignancies generally occur from either localised progression of disease, radiotherapy, or prior surgical intervention [1].

The most common types of fistula are enterovaginal and vesicovaginal fistulas, however many other types can occur due to the number of organs and vessels in close proximity to each other in the pelvic space.

Different treatment options are available depending of the site of the fistula, however much consideration has to be given to the physical status of the patient as well as the affected diseased tissue. Certain treatment options, such as surgery to cover/close the fistula or surgical diversion may not be appropriate due to the fragility of the underlying tissue or from underlying patient comorbidities. Conservative management with simple diversion of the resulting leaking material may not be appropriate due to (1) the complexity of the fistula and involved organs, (2) the associated risk of infection, and (3) the resulting effect for the patient of having a permanent catheter via urethra or rectum.

Interventional radiology plays an important role in the therapeutic and palliative treatment of many types of advanced cancers. Interventional procedures performed for treatment of fistulas, such as the one discussed in this case report, can be done with minimally invasive techniques, without the need for anaesthetic intervention, and take into account patient factors.

This work has been reported in line with the SCARE criteria [2].

## 2 Case presentation

A 37 year-old female with recurrent metastatic cervical squamous cell carcinoma previously treated with chemotherapy and radiotherapy, presented to her oncology team with increasing watery discharge per vagina. The patient was known well known and under the care of oncology in the Irish tertiary academic centre. The patient had recently had a left ureteric stent placed for new left hydronephrosis. The patient had no prior medical history, and lived locally with her partner.

### 2.1 Clinical findings

Clinical examination demonstrated some mild abdominal and pelvic tenderness. Fluid was noted to be discharging per the vagina.

### 2.2 Timeline

The patient had noted foul smelling watery discharge per vagina over a period of three months, which was increasing in frequency with associated reduced urinary output.

### 2.3 Differential diagnosis

Given the known encasement and involvement of the left ureter by the cervical mass and hydronephrosis, a ureterovaginal fistula was felt the most likely diagnosis. A vesicovaginal fistula was another differential diagnosis.

### 2.4 Diagnostic assessment

An MRI pelvis demonstrated interval disease progression with the large left side predominant parametrial mass invading the posterior bladder wall, rectum and vagina, and encasing the distal ureter and stent with a direct ureterovaginal fistulation (Fig. A).

**Fig. A:**
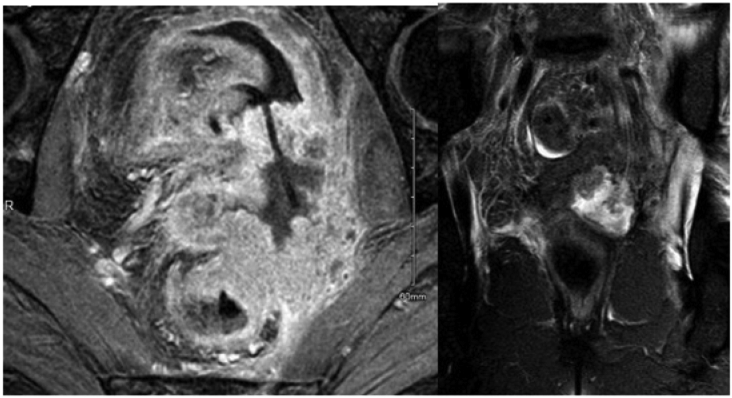
Axial and coronal MRI sequences demonstrating locally advanced parametrial tumour invading the urinary bladder wall, rectum and vagina. Complete encasement of the distal left ureter with large uretero-vaginal fistula. Left ureteric stent in situ.

### 2.5 Therapeutic intervention

An initial decision was made to insert bilateral nephrostomies with the aim of urinary diversion from the uretero-vaginal fistula, however this had a limited effect, and the patient continued to experienced leaking urine from the vagina.

The patient's case was re-discussed with interventional radiology, and the decision made to perform ureteric embolization of the left ureter, which was the main site of fistulation, in order to stop urine leaking. This warrants permanent urinary diversion via nephrostomy following the procedure. As the patient had a pre-existing left ureteric stent, a novel fluoroscopic-guided transurethral retrograde stent removal and ureteric embolization was planned. This allowed the patient to remain supine for the entire procedure, avoiding the inconvenience of transferring from prone to supine, which would be necessary for conventional stent removal and antegrade ureteric embolisation. Furthermore, given the patient's advanced disease, prone position caused significant discomfort.

#### 2.5.1 Procedure

Under fluoroscopic guidance, the bladder was accessed with a guidewire via a urinary catheter, which was subsequently removed. A catheter was placed over the wire and a snare was used via the catheter to partially withdraw the distal pigtail of the stent to the level of the urethra. A guidewire was advanced through the stent to secure access of the ureter and the stent fully removed. Coils were placed via a Bernstein catheter in the mid ureter and within the distal ureter at the site of fistulisation. Cessation of flow through the ureter was demonstrated by performing a left nephrostogram (Fig. B).

**Fig. B:**
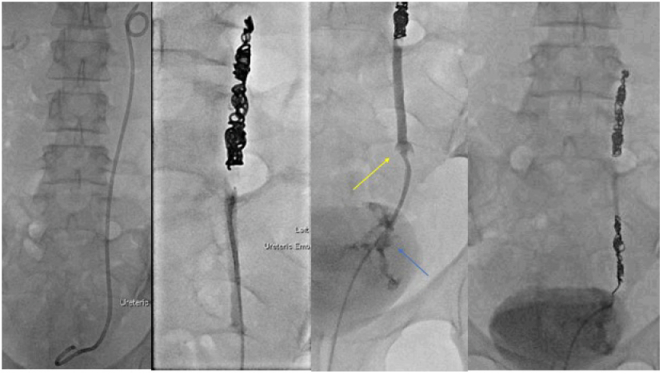
Transurethral removal of left ureteric stent and retrograde ureteric coiling. Fluoroscopic images demonstrate bilateral nephrostomies and left ureteric stent in situ. Transurethral snaring of left ureteric stent allows for partial withdrawal, insertion of wire to left ureter and removal of stent over wire. A Bernstein catheter is inserted over wire and metallic coils deployed in mid ureter. A filling defect is seen in the distal left ureter (yellow arrow) with contrast leak to vagina (blue arrow), consistent with tumour encasement and fistula. Additional coils were placed at site of defect. (For interpretation of the references to colour in this figure legend, the reader is referred to the web version of this article.)

Antegrade embolization of the right ureter was performed the following day via the right nephrostomy. This was the only option for access as there was no right ureteric stent. An Amplatz plug was placed in the mid-ureter via a 7F sheath. Multiple coils were then deployed via a Bernstein catheter above the plug. A right nephrostogram demonstrated minimal flow within the right ureter post embolisation (Fig. C).

**Fig. C:**
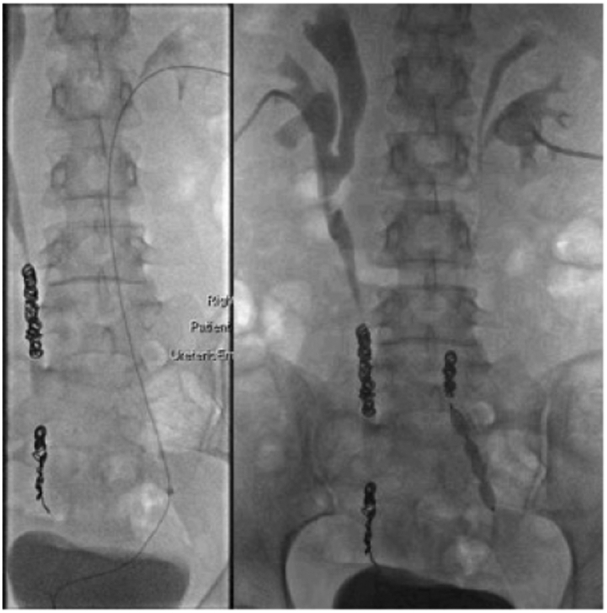
Antegrade right ureteric coiling. The right ureter is accessed with guidewire via right nephrostomy. A 7Fr sheath was advanced over wire and an Amplatzer plug was deployed via the sheath in the distal right ureter. Coils were deployed via Bernstein catheter in the mid right ureter. Post-coiling bilateral nephrostograms demonstrate limited flow in ureters with no contrast advancing to bladder. Partial duplex collecting systems with high confluences bilaterally.

### 2.6 Follow up and outcomes

The patient experienced a significant decrease in the volume of vaginal discharge and discomfort post embolisation and was discharged from hospital shortly after. The patient was followed up by her primary oncology team in the oncology day ward clinic and through her local community palliative care team. No further intervention was required by radiology.

## 3 Discussion

Interventional radiology plays an integral role in symptom management in end-stage disease. Given that many patients may not be suitable for surgical or simple conservative management due to the complexity of the fistula and their advanced disease, other non-surgical interventional measures are required in order to successfully manage a patient's symptoms and reduce the risk of medical complications, such as infection. A creative and sometimes novel approach may need to be taken in order to provide a solution for a problem that otherwise will likely have a non-successful treatment outcome [3].

Permanent ureteric embolization may be considered as a palliative procedure to facilitate urinary diversion via nephrostomy in cases of ureteric, urethral, or bladder fistulation [4]. Patients considered for this approach, such as the patient discussed in this case report, are those who present towards the end of their disease and require a palliative approach in order to gain symptom control and improve quality of life.

The resulting symptoms from fistulisation, particularly if they involve colonic or ureteric structures can be particularly distressing for the patient [5]. Both the physical and psychological effect from vaginal fistulation are an unnecessary burden for patients who are generally at the end stage of their disease.

Prolonged immobilisation in the prone position can be uncomfortable for such patients due to other sequalae of their disease such as ascites and general abdominal discomfort. A retrograde transurethral approach is a novel technique for those with existing ureteric stents, and is particular useful for patients with a ureterovaginal fistula allowing for stent removal and ureteric embolization through single access site in a supine position, making the procedure easier for both the patient and the radiologist.

## 4 Conclusion

Localised pelvic fistulisation secondary to advanced gynaecological malignancy is a not uncommon complication that can have serious physical as well as psychological complications for patients, both of which are additional burdens on a patient at the end stage of their disease.

Interventional radiology plays an important role in palliative and symptomatic management of end stage malignant disease. Ureteric embolisation via a retrograde transurethral approach by way of an existing stent is a novel approach to access making the procedure easier for both the patient and radiologist.

## Patient consent

Written informed consent was obtained from the patient for publication of this case report and the accompanying images. A copy of the written consent is available for review by the Editor-in-Chief of this journal on request.

## Funding

None.

## Ethical approval

Case report – patient consent granted.

## Guarantor

Holly Acton.

## Research registration number

Not applicable.

## CRediT authorship contribution statement

**H. Acton:** Writing – original draft. **R. Motyer:** Writing – review & editing. **J. O'Mahony:** Writing – original draft. **I. Brennan:** Supervision. **J.M. Ryan:** Supervision.

## Declaration of competing interest

The authors have no conflicts of interest to declare.
